# MIND-OUT: Medications in Intensive Care, Delirium and OUTcomes

**DOI:** 10.1192/bjo.2025.10209

**Published:** 2025-06-20

**Authors:** Hannah Reynolds, Bhamini Patel, Sara Ormerod, Felicity Evison, William Tunnicliffe

**Affiliations:** 1 Birmingham and Solihull Mental Health NHS Foundation Trust, Birmingham, United Kingdom; 2 Coventry and Warwickshire Partnership NHS Trust, Coventry, United Kingdom; 3 University Hospitals Birmingham NHS Foundation Trust, Birmingham, United Kingdom

## Abstract

**Aims:** To investigate how anticholinergic burden of medications changes during hospital stay for Intensive Care Unit (ICU) patients and to review whether anticholinergic burden predicts delirium and mortality.

Delirium is a common cause of morbidity and mortality within ICU. Anticholinergic Burden (ACB) and Anticholinergic Effect on Cognition (AEC) tools are validated to assess anticholinergic effects from medication. Scores of ≥3 are associated with increased delirium and mortality. This study investigates anticholinergic burden from ICU admission through to hospital discharge.

**Methods:** Retrospective, ethically approved study of adults (N= 6,305) admitted to ICU in University Hospitals Birmingham over 3 years (2021–2023). Subjects were excluded if <48 hours spent in ICU or readmissions (within a year). Both ACB and AEC tools were used to assess anticholinergic burden (AEC is more sensitive to the cognitive effects of medications). Timepoints assessed: ICU admission, ICU discharge, hospital discharge, and maximum score. We explored secondary outcomes including delirium and mortality. Research performed in collaboration with PIONEER (Health Data Research Hub for Acute Care).

**Results:** Median age 60.0 years, 61.9% male, and 71.6% of white ethnicity. Median time from hospital to ICU admission 17.1 hours, 86.3% emergency admissions. Median length of stay in ICU 5.2 days (19.2 days in hospital).

Difference in mean score from ICU admission to ICU discharge was +0.38 (p<0.001) for ACB, and +0.29 (p<0.001) for AEC; from ICU discharge to hospital discharge was −0.12 for ACB (p<0.001) and +0.36 (p=0.005) for AEC.

There was a significant rise in patients with high-risk scores (ACB or AEC ≥3): admission to ICU 9.9% had ACB ≥3, and at discharge from ICU 19.9% (p<0.001), with no significant fall back at hospital discharge (18.9%, p=0.229). The AEC tool showed similar **Results:** admission to ICU, 4.9% of patients had AEC ≥3 and at discharge from ICU 10.5% (p<0.001) However, this tool showed a further rise by hospital discharge 12.3% (p<0.003). Delirium was inadequately recorded.

Results showed anticholinergic burden significantly increases following ICU admission. The proportion of patients with high-risk scores doubles during ICU admission and does not significantly reduce prior to discharge. We were unable to link these scores to delirium with insufficient data collected, but investigations on possible links to mortality are ongoing.

**Conclusion:** This study demonstrated a significant rise in anticholinergic burden linked to ICU admission, persisting to hospital discharge. This places such patients at risk of drug-induced morbidity and mortality.

